# Hemorrhagic Pericardial Effusion and Cardiac Tamponade in B-chronic Lymphocytic Leukemia Treated With Ibrutinib: A Case Report

**DOI:** 10.7759/cureus.77872

**Published:** 2025-01-23

**Authors:** James C Barton, Luigi F Bertoli, James B Cavender

**Affiliations:** 1 Department of Medicine, The University of Alabama at Birmingham, Birmingham, USA; 2 Department of Medicine, Brookwood Baptist Medical Center, Birmingham, USA

**Keywords:** bruton tyrosine kinase, hemostasis, ibrutinib, platelet, pleural effusion, serositis

## Abstract

There are few reports of non-malignant hemorrhagic pericardial effusion and cardiac tamponade in patients with B-chronic lymphocytic leukemia (B-CLL) who were not concurrently treated with an anticoagulant or an anti-platelet agent. We report the case of a 57-year-old woman with B-CLL treated for 54 months with single-agent ibrutinib who developed non-malignant hemorrhagic pericardial effusion and cardiac tamponade. She recovered after pericardiocentesis and the discontinuation of ibrutinib treatment. We conclude that the pathogenesis of her hemorrhagic pericardial effusion involved a combination of abnormal coagulation and platelet dysfunction typical of B-CLL, off-target kinase effects of ibrutinib on platelets, and serosal inflammation.

## Introduction

Ibrutinib, an orally administered, irreversible inhibitor of Bruton tyrosine kinase (BTK) (XLA; chromosome Xq22.1), is licensed in the U.S. for treatment of adults with B-chronic lymphocytic leukemia (B-CLL)/small-cell lymphocytic lymphoma, mantle cell and marginal zone lymphomas, and Waldenström macroglobulinemia, malignancies of B-lymphocytes that occur predominantly in middle-aged or older adults. Ibrutinib covalently binds to the cysteine 481 residue in the adenosine triphosphate (ATP) binding site of BTK, a member of the Tec kinase family [1,2]. Ibrutinib also binds to other Tec kinases such as ITK [3] and ErbB family kinases such as epidermal growth factor receptor (EGFR) and human epidermal growth factor receptor 2 (HER2) [4], all of which have a cysteine residue at the homologous active site.

Treatment of B-CLL with ibrutinib was associated with bleeding-related adverse events (defined as any patient-reported bleeding or bruising, or any documentation of bleeding or bruising upon physical examination) of grade ≤2 severity in 55.3% of 85 patients (and infrequently with events of grade ≥3 severity) in one study [5]. In another study, one of 364 patients with B-CLL treated with ibrutinib developed a pericardial effusion not otherwise specified [6]. In separate case reports, two women with B-CLL treated with ibrutinib developed non-malignant hemorrhagic pericardial effusion and cardiac tamponade [7,8]. Hemorrhagic pericardial effusions and cardiac tamponade have also been reported in patients with B-CLL treated with ibrutinib and a concurrent anticoagulant [9,10] or anti-platelet agent [11].

We report the case of a 57-year-old woman with B-CLL treated with single-agent ibrutinib for 54 months who developed non-malignant hemorrhagic pericardial effusion, cardiac tamponade, and pleural effusions without concurrent treatment with an anticoagulant or an anti-platelet agent. We discuss risk factors for bleeding-related adverse events and pericardial effusions including abnormal coagulation, platelet dysfunction, and inflammation in patients with B-CLL treated with or without ibrutinib.

## Case presentation

A 50-year-old asymptomatic White woman was evaluated for lymphocytosis (day -2526). She reported having five episodes of sinusitis a year. She took no regular prescription medications. Her son and daughter had recurrent sinusitis. Her mother died at age 87 years of chronic lymphocytic leukemia.

Physical examination revealed no significant abnormality. Laboratory values are displayed in Table 1. Serum protein electrophoresis and immunofixation were normal. Flow cytometry of blood mononuclear cells detected a monoclonal B-cell population that variably expressed CD19, CD20, CD11c, CD23, and CD5 without CD38 co-expression. Fluorescent in situ hybridization revealed homozygosity for a 13q14 deletion and normal results for CCND1/IGH, ATM, 12cen, and TP53. ZAP-70 was negative. These data were interpreted as B-CLL (Rai stage 0) and subnormal IgG3/IgM. In patients with B-CLL, the absence of hypogammaglobulinemia G, monoclonal IgM, and ZAP-70 positivity and the presence of 13q14 deletion are associated with favorable progression-free survival and time to treatment.

**Table 1:** Laboratory values in a woman with B-chronic lymphocytic leukemia ALC: absolute lymphocyte count; ALT: serum alanine aminotransferase; ANC: absolute neutrophil count; AP: alkaline phosphatase; AST: serum aspartate aminotransferase; CRP: C-reactive protein; ESR: erythrocyte sedimentation rate; Ig (serum): immunoglobulin; nd: not done; WBC: white blood cells Day 0 was defined as the date on which the patient presented with manifestations interpreted as pericardial effusion and cardiac tamponade. Other results on day 0 specimens included prothrombin time 11.4 s (INR 1.07; reference range 11.0-13.5 s), free thyroxine 18.28 pmol/L (reference range 10.30-21.88 pmol/L), and thyroid-stimulating hormone 3.44 µIU/L (reference range 0.450-4.500 µIU/L). Negative results included blood and urine cultures; nucleic acid analyses for multiple bacteria in blood; rapid antigen testing for SARS-CoV-2; and serologic testing for viral hepatitis B and C. The negative results of these latter tests substantiated that it was unlikely that an infectious disease accounted for cardiac tamponade and elevated serum levels of hepatic transaminases.

Day	-2526	-1550	-1107	-51	0	1	2	3	4	14	40	186	Reference range
Hemoglobin, g/L	132	117	123	134	128	110	107	101	108	134	121	134	120-180
WBC x 10^6^/L	29.2	62.8	10.3	10.0	33.2	24.4	18.5	11.8	11.2	16.0	8.4	6.6	4.1-10.9
ANC x 10^6^/L	5.1	10.2	5.4	4.3	22.6	15.5	11.1	6.5	nd	2.7	5.3	3.4	2.0-7.8
ALC x 10^6^/L	22.7	45.2	4.3	5.1	6.3	5.6	5.1	3.5	nd	12.3	2.5	2.6	0.6-4.1
Platelets x 10^6^/L	183	172	167	185	562	443	426	368	366	300	251	199	140-440
IgG1, g/L	5.03	nd	nd	nd	nd	nd	nd	nd	nd	nd	nd	nd	4.22-12.92
IgG2, g/L	1.74	nd	nd	nd	nd	nd	nd	nd	nd	nd	nd	nd	1.17-7.47
IgG3, g/L	0.04	nd	nd	nd	nd	nd	nd	nd	nd	nd	nd	nd	0.41-1.29
IgG4, g/L	0.18	nd	nd	nd	nd	nd	nd	nd	nd	nd	nd	nd	0.01-2.91
IgA, g/L	1.08	nd	nd	nd	nd	nd	nd	nd	nd	nd	nd	nd	0.91-4.14
IgM, g/L	0.18	nd	nd	nd	nd	nd	nd	nd	nd	nd	nd	nd	0.40-2.30
ALT/AST, IU/L	nd	nd	nd	13/21	75/47	47/27	nd	44/30	nd	21/21	nd	24/18	0-32/0-40
AP, IU/L	nd	nd	nd	92	401	241	nd	289	nd	233	nd	76	44-121
Bilirubin, µmol/L	nd	nd	nd	10.3	35.9	17.1	nd	10.3	nd	8.6	nd	8.6	1.7-20.5
Lactate, mmol/L	nd	nd	nd	nd	3.7	1.1	0.9	nd	nd	nd	nd	nd	<2.0
Troponin-I, ng/L	nd	nd	nd	nd	19	nd	nd	nd	nd	4	nd	nd	<47
CRP, mg/L	nd	nd	nd	nd	317	nd	nd	nd	nd	67	nd	nd	0-10
ESR, mm/h	nd	nd	nd	nd	57	nd	nd	nd	nd	25	nd	nd	0-20

She developed progressive fatigue and lymphocytosis (Table 1, day -1550) during an observation interval of 32 months. Treatment with ibrutinib 420 mg daily was prescribed to alleviate these manifestations. After 15 months of ibrutinib treatment, her fatigue and lymphocytosis (Table 1, day -1107) had resolved. She continued ibrutinib treatment for an additional 39 months, remained asymptomatic, and had stable CBC and normal chemistry profile values (Table 1, -day 51).

At age 57 years, she presented to an emergency department (day 0) with a one-week history of chest fullness, abdominal pain, nausea, emesis, diarrhea, prostration, and moderate distress. She had discontinued ibrutinib treatment due to these manifestations. The annual tuberculin skin tests required by her employer had been negative. She had tachycardia and mild abdominal tenderness. Blood test results are displayed in Table 1. CT and CT angiography (CTA) images of the chest with contrast revealed a large pericardial effusion, a moderate left pleural effusion with compressive atelectasis, trace right pleural effusion, and no mass. There was no pulmonary thromboembolism. CT images of the abdomen and pelvis and abdominal ultrasonography also revealed pleural effusions and no mass. Enlarged lymph nodes and splenomegaly were not detected in any image. Hematologic and radiographic evidence at presentation with pericardial effusion and cardiac tamponade indicated that the B-CLL of the present woman was in remission.

**Figure 1:**
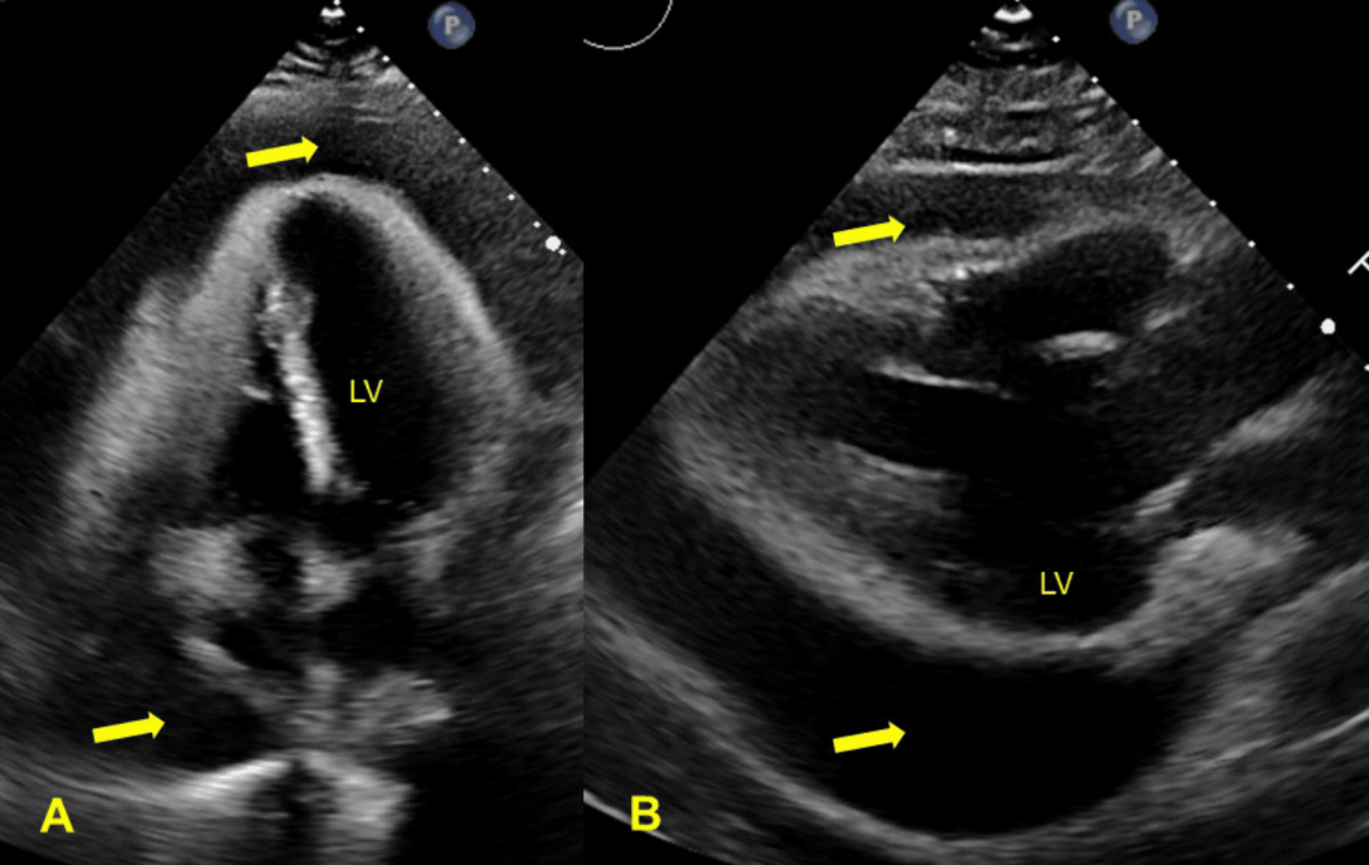
Transthoracic echocardiograms at emergent presentation (day 0) Large circumferential pericardial effusion is seen in (A) the apical four-chamber view (arrows) and (B) the parasternal long-axis view (arrows). LV: left ventricle

An electrocardiogram and cardiac monitoring detected atrial fibrillation which was resolved with diltiazem administration. Echocardiograms detected a large pericardial effusion, phase I cardiac tamponade, and a left ventricular ejection fraction of 60-65% (Figure 1). Pericardial effusion with cardiac tamponade, together with the available laboratory results and discovery of similar manifestations in reports of other patients with B-CLL, was interpreted as an adverse effect of ibrutinib therapy.

Pericardiocentesis on day 0 yielded 720 mL of hemorrhagic fluid with red blood cells 107 x 10^6^/L, WBC 6.3 x 10^6^/L (96% neutrophils, 4% mononuclear cells), pH 7.5, protein 4.7 g/L, lactate dehydrogenase 777 IU/L, and glucose 4.44 mmol/L. Gram stain, cultures, and nucleic acid analyses of the fluid for pyogenic bacteria and *Mycobacterium tuberculosis *were negative. Her symptoms, neutrophilia, thrombocytosis, and elevated serum liver enzyme and bilirubin levels abated (Table 1). She was discharged from the hospital on day 5 to take diltiazem 180 mg daily and colchicine 0.6 mg twice daily and to wear a cardiac monitor for three weeks. An echocardiogram on day 14 revealed a small circumferential pericardial effusion.

Light transmission platelet aggregometry was performed on day 40 when the patient had not taken ibrutinib for more than 40 days nor aspirin, clopidogrel, or other adenosine diphosphate (ADP) receptor inhibitors. The patient's platelets did not respond to collagen or ADP. Platelet aggregation and ATP release in response to thrombin and arachidonic acid were normal.

**Figure 2:**
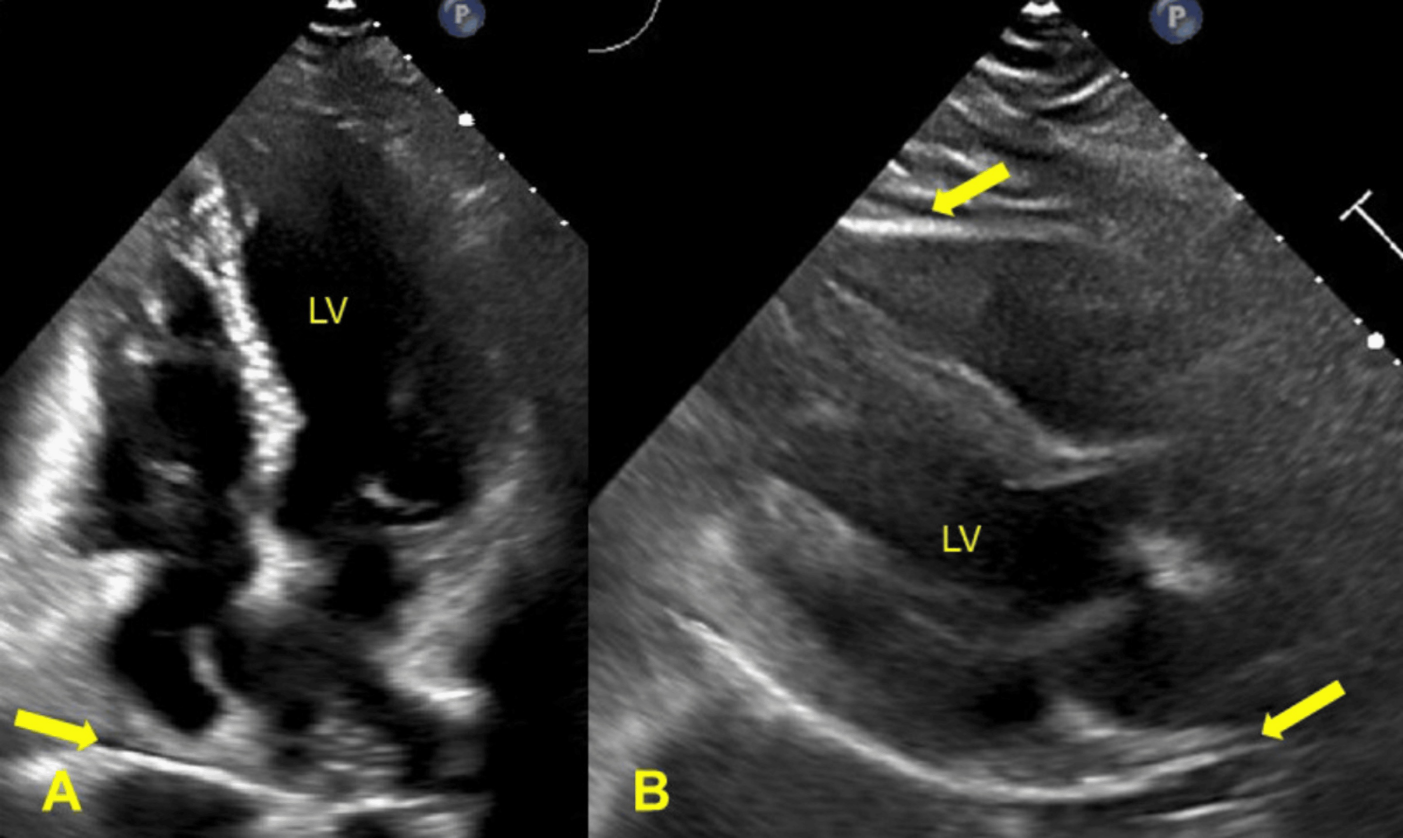
Transthoracic echocardiograms after treatment (day 68) Physiologic circumferential pericardial fluid is seen in (A) the apical four-chamber view (arrow) and B) the parasternal long-axis view (arrows) after pericardiocentesis and discontinuation of ibrutinib therapy. LV: left ventricle

On day 68, her echocardiogram was normal (Figure 2) and a one-week Holter monitor recording revealed no arrhythmia. On day 186, she felt and appeared normal off-therapy for B-CLL. CBC and other measures were within respective reference limits (Table 1). There was no indication of initiating second-line therapy for B-CLL.

## Discussion

Median age, treatment indication, Rai leukemia stage, and median platelet count at diagnosis in a prospective study did not differ significantly in 47 patients who did (55.3%) and 38 patients who did not (44.7%) experience bleeding-related adverse events after initiation of single-agent ibrutinib treatment of B-CLL [5]. The aggregate relative risk of bleeding-related adverse events in 23 patients with B-CLL (27.1%) who also took aspirin, other non-steroidal anti-inflammatory drugs, fish oil, dabigatran, or apixaban was not significantly elevated [5]. These observations suggest that the general baseline characteristics of patients with B-CLL do not predict increased risks of bleeding-related adverse events that occur after initiation of ibrutinib treatment.

BTK is normally expressed in B-lymphocytes and platelets [12]. Persons with X-linked agammaglobulinemia (XLA) have severe BTK deficiency, the underlying cause of their almost total absence of B-lymphocytes and plasma cells. Although individuals with XLA lack platelet BTK, they do not have a bleeding disorder [12]. Thus, it is unlikely that the irreversible binding of ibrutinib to platelet BTK causes adverse bleeding-related events in patients with B-CLL.

Coagulation measures present before ibrutinib treatment, including prolonged epinephrine closure time, lower levels of von Willebrand factor activity, and lower levels of Factor VIII, were associated with a significantly increased risk of bleeding-related adverse events that eventually occurred in 47 of 85 patients (55.3%) with B-CLL [5]. Platelet function measured with a platelet function analyzer was impaired in 22 patients (25.9%) with B-CLL before ibrutinib treatment [5]. These observations indicate that patient- or leukemia-specific factors account in part for increased risks of bleeding-related adverse events in patients with B-CLL.

Light transmission platelet aggregometry in the present patient performed more than 40 days after she discontinued taking ibrutinib detected a lack of platelet reactivity to collagen, typical of untreated patients with B-CLL [13], and a lack of platelet reactivity to ADP. Thus, it is plausible that this patient's platelets were unreactive to both collagen and ADP before or during ibrutinib treatment, although her platelet aggregation was not studied before or during ibrutinib treatment.

Pharmacologic concentrations of ibrutinib selectively inhibited collagen-induced platelet aggregation in platelet-rich concentrates from healthy blood donors [14] and patients taking ibrutinib therapy [15]. Off-target inhibition of platelet Tec kinases by ibrutinib [2] may account in part for bleeding-related adverse events in patients with B-CLL treated with ibrutinib. Together, these observations suggest that ibrutinib treatment increases the risk of bleeding-related adverse events in some patients with B-CLL who do not take concurrent anticoagulants or anti-platelet agents. Ibrutinib primarily affects collagen-dependent platelet aggregation and thus this can be monitored when assessing platelet function in patients before and during ibrutinib therapy.

Serositis with consequent pericardial and pleural effusions has been described in patients treated with ibrutinib for B-CLL [16], mantle cell lymphoma [17], and Waldenström macroglobulinemia [18]. The mechanism(s) underlying the development of serositis in these patients is incompletely understood. In the present woman, pericardial fluid was exudative, although we did not obtain a specimen of the pericardium or pleural fluid for histologic analysis. Diverse viruses including Epstein-Barr virus and cytomegalovirus cause pericarditis and pleuritis, although the present woman did not report having a "flu-like" prodrome nor was she tested for viral infection other than that caused by SARS-CoV-2 and hepatitis B and C. It is plausible but unproven that immune dysregulation or mesothelial injury contributes to the pathogenesis of serositis in patients treated with ibrutinib.

Leukemic infiltration of the pericardium is a rare cause of hemorrhagic pericardial effusions and cardiac tamponade in patients with previously untreated B-CLL [19]. In the present woman, there was a relative paucity of lymphocytes in blood and pericardial fluid. Enlarged lymph nodes and splenomegaly were not detected by CT scanning. These observations exclude the possibility that she had leukemic infiltration of the pericardium and substantiate that specific therapy for leukemic pericardial infiltration was not indicated.

The risk of developing recurrent or chronic atrial fibrillation is increased in patients with B-CLL treated with ibrutinib [6]. In the present woman, we attributed transient atrial fibrillation to cardiac tamponade. Transient atrial fibrillation also occurred in another woman treated with ibrutinib who developed non-malignant hemorrhagic pericardial effusion and cardiac tamponade without concurrent treatment with an anticoagulant or an anti-platelet agent [8].

Decreased cardiac output due to cardiac tamponade causes hepatic venous congestion and ischemic liver injury [20]. These factors would account for this patient's elevated serum concentrations of hepatic enzymes and bilirubin that abated after pericardiocentesis.

## Conclusions

Bleeding-related adverse events of grade ≤2 severity are common in patients with B-CLL treated with ibrutinib. Many patients with previously untreated B-CLL have abnormal coagulation and platelet dysfunction that may become more severe with ibrutinib treatment, especially patients who also take anticoagulation or anti-platelet therapy for non-leukemia indications. Bleeding-related adverse events not explained by age, treatment indication, Rai leukemia stage, subnormal platelet count, ibrutinib binding to platelet BTK, or anticoagulation or anti-platelet therapy for non-B-CLL indications are probably due to abnormal coagulation or platelet dysfunction typical of untreated B-CLL or the off-kinase effects of ibrutinib treatment on platelets. Some patients with B-CLL develop serositis during ibrutinib treatment. We conclude that (1) the pathogenesis of non-malignant hemorrhagic pericardial effusion, cardiac tamponade, and pleural effusions that occurred in the present woman with B-CLL treated with single-agent ibrutinib for 54 months probably involved a combination of abnormal coagulation and platelet dysfunction typical of B-CLL, off-target kinase effects of ibrutinib, and serosal inflammation and that (2) it is contra-indicated to treat a recurrence of B-CLL in this patient with a BTK inhibitor. Coagulation testing and platelet aggregometry should be considered in patients with B-CLL before and during ibrutinib therapy.
